# The Association between Organizational Justice and Psychological Well-Being by Regular Exercise in Korean Employees

**DOI:** 10.3390/ijerph16122223

**Published:** 2019-06-24

**Authors:** Hanul Park, Kang-Sook Lee, Yong-Jun Park, Dong-Joon Lee, Hyun-Kyung Lee

**Affiliations:** 1 Department of Preventive Medicine, College of Medicine, The Catholic University of Korea, 222 Banpo-daero, Seocho-gu, Seoul 06591, Korea; onefence@catholic.ac.kr; 2 Department of Public Health, Graduate School, The Catholic University of Korea, 222 Banpo-daero, Seocho-gu, Seoul 06591, Korea; 3 Graduate School of Public Health, The Catholic University of Korea, 222 Banpo-daero, Seocho-gu, Seoul 06591, Korea; jun14t@catholic.ac.kr (Y.-J.P.); dj3879@hanmail.net (D.-J.L.); lhk701@naver.com (H.-K.L.)

**Keywords:** exercise, interactional justice, Korea, lifestyle, procedural justice, psychological well-being

## Abstract

Many studies have shown that organizational justice (OJ) is related to psychological determinants of employees’ physical and mental health in the workplace, and these health outcomes also lead to the psychological well-being (PW) of employees. Additionally, physical activity is one of the most important issues related to health in the workplace. This study compared the level of perceived OJ according to sociodemographic and lifestyle factors and examined the association between OJ and PW by regular exercise (hours per week) in Korean employees. This study used cross-sectional data obtained from 494 subjects in South Korea. Self-administered questionnaires comprising OJ, PW, and lifestyle factors (e.g., smoking, drinking, sleeping, and exercise) were completed by employees in April 2017. Multiple logistic regression analyses were conducted to estimate the association of procedural justice (PJ) and interactional justice (IJ) with the prevalence odds ratios and 95% confidence intervals of the high risk to PW. After the adjustment of sociodemographic characteristics and lifestyle factors, the main effects of PJ and IJ on the high risk to PW were significantly observed, and when these values were stratified by a regular exercise category, the lowest odds ratio was observed in a group that exercised for 1–2 h (hours per week). Organizations must encourage trust and consideration between employees and supervisors and carry out efforts to improve their environment, such as making the decision-making process fairer and encouraging employees to exercise regularly. This intervention may help prevent a high risk to PW.

## 1. Introduction

Organizational justice (OJ), which can be defined as the employees’ belief that supervisors are considering their viewpoints, sharing information about decision-making, and treating them fairly and truthfully, is a measure of justice in the workplace [1]. The interaction between employees and supervisors at work is a very important factor and is highly relevant to OJ. Furthermore, studies have reported that OJ is a determinant of physical and mental health [2,3]. To be specific, the concept of OJ is related to employees’ perception of fair treatment at the workplace [4]. It can be further classified into procedural justice (PJ) (whether the opinions of employees affected by the decision-making process are consistently applied, accurate, correctable, unbiased, and ethical), interactional justice (IJ) (whether supervisors and employees treat each other with respect and consideration, and how well the rationale for decisions is explained when procedures are implemented), and distributive justice (DJ) (whether outcomes are consistent with implicit norms for allocation, such as equity or equality) [5,6]. Our study focused on the PJ and the IJ.

Many studies have reported on the relationship between health and justice. A recent meta-analysis study of 290 public workers in the United States found an association between OJ and cardiovascular health. In particular, the study showed that lowering the heart rate and systolic and diastolic blood pressure in the workplace has a relationship to a high level of PJ and perceived organizational support [7]. And low PJ was associated with an increased risk for psychiatric disorders, sickness absence, and psychological distress [8,9,10]. Also, a recent prospective cohort study that observed employees in Japan for one year found that interactional justice and the onset and persistence of insomnia were related [11]. The perceived level of OJ according to sociodemographic and lifestyle variables has been studied in Japan [12] and Taiwan [13]. However, we also compared the level of OJ according to sociodemographic and lifestyle factors in Korean employees because East Asian countries such as Korea, Japan, and China have substantially different attitudinal patterns than in other parts of the world, with characteristics of high collectivism and power distance [14].

Psychological well-being (PW) can be related to self-esteem, cognitive function, personality, and mood, including positive influences such as happiness, vigor, and morale, and negative influences such as anxiety and depression [15]. The World Health Organization recognizes well-being as an important factor of health that plays an important role in employees’ and supervisors’ relationships as well as in job satisfaction and productivity relationships in the workplace [16,17].

Recent studies on the relationship between OJ and health outcomes have focused on the effect of various factors: age, enterprise size, smoking status, employment contract status, and six types of coping behaviors (active solutions, seeking help for solutions, changing moods, emotional expression involving others, avoidance and suppression, and changing points of view) [4,12,13,18]. In the Japan study, the association of low level of PJ with serious psychological distress was more likely to be harmful to smokers than to non-smokers [12]. These preceding studies have examined lifestyle modification effects, and Nagakawa [18] proposed coping strategies focusing positive emotion such as changing a point a view as an alternative to the negative relationship between OJ and psychological distress. Many studies [12,19] have reported on the effects of organizational justice on health in the workplace, but whether its effects differ by lifestyle variables such as exercise has hardly been examined. 

Physical activity is one of the most important issues related to mental and physical health in the workplace [20,21,22], but many countries have adopted Westernized lifestyles that are associated with a decline in physical activity primarily due to mechanization and automation of occupational activities. A recent study in the Netherlands found that physical activity was associated with a better health-related quality of life (HRQL) [23]. Regular exercise has been characterized as a health behavior related to physiological benefits [24], and this has also been documented to include many other psychological benefits such as a reduction of depressive mood [25,26] and anxiety [27]. We classified hours of regular exercise according to hours of exercise for improving health in daily life based on the ‘Exercise guidelines for Health Promotion’ [28] and did not distinguish between exercise in free time and in the workplace. In a study conducted in Finland [24], a total of 3403 subjects were observed to explore the association between exercise frequency and PW. The Finland study showed a consistent association between enhanced PW and regular exercise (defined as frequency of exercise during spare time). These participants also experienced a stronger feeling of social integration than those who exercise less frequently exercisers did [24]. In addition, physical activity was associated with improved physiological functioning, maintenance of normal body weight and blood pressure, maintenance of level of plasma lipid and lipoprotein levels, and self-concept [29,30]. The U.S. Department of Health and Human Services highlighted that physical exercise and fitness are considered vital to promoting mental health [31]. Regular exercise in employees could help alleviate musculoskeletal stress experienced at work, which could lead to improved productivity in employees. It could also act as a morale booster for employees who may subsequently view management as being actively involved in their well-being [32]. The World Health Organization emphasizes the promotion of physical activity in the workplace as a key intervention to achieve better health and well-being in the working population [33]. But it is not easy for workers to make time for exercise in an increasingly busy society. The data from Korea indicate that the rate of aerobic and strength exercise practiced more than twice a week is approximately 20% in men and 10% in women [34]. We estimated that the association between OJ and PW in the workplace will vary by the frequency of regular exercise.

The purpose of the present study was to compare the perceived level of OJ by sociodemographic factors and lifestyle factors and examine the association between OJ and PW by regular exercise (hours per week) in Korean employees.

## 2. Materials and Methods

### 2.1. Participants

We obtained data from 494 subjects employed by enterprises of varying size in Seoul and Gyeonggi Province, South Korea: 182 from large enterprises, 169 from medium enterprises, and 143 from small enterprises. Enterprise size was classified into large (more than 300 employees), medium (50–299 employees), and small (1–49 employees). Self-administered questionnaires were delivered either directly or by mail and were completed by employees in April 2017. Employees whose responses were missing in the questionnaires were excluded (118), thus, a total of 376 employees (142 from large enterprises, 131 from medium enterprises, 103 from small enterprises) were finally analyzed. The gender of the participants was 219 men (58.2%) and 157 women (41.8%). In type of employment, regular was 298 (79.3%) and temporary was 78 (20.7%). The Institutional Review Board at the Catholic University of Korea reviewed and approved the design of this study (IRB approval number: MC18QESI0026).

### 2.2. Measurements

#### 2.2.1. Organizational Justice (OJ)

The Korean version of the Organizational Justice Questionnaire (K-OJQ) was used to measure PJ and IJ [35]. K-OJQ consisted of seven items for PJ and six items for IJ. The responses to each item were recorded on a five-point Likert scale (range: 1 = strongly disagree; 2 = disagree, 3 = neither agree nor disagree, 4 = agree, 5 = strongly agree). The K-OJQ, which was translated from a modified version of Moorman’s OJQ [36] and the Japanese version of the OJQ [6], was developed by conducting a preliminary survey and through translation–back translation. The internal consistency reliability factor and construct validities of the K-OJQ have been shown to have acceptable values [35]. In this sample, Cronbach’s alpha coefficients were 0.92 for PJ and 0.94 for IJ.

#### 2.2.2. Psychological Well-Being (PW)

PW was measured by the Psychological Well-Being Index—short form (PWI-SF) [37], which was based on Goldberg’s General Health Questionnaire (GHO60) [38], and was developed to standardize the stress measurement of Koreans. The PWI consists of four factors: social role performance and self-reliability, depressive mood, general health and vitality, and sleep disturbance and anxiety. Items were scored using a 4-point Likert scale (range: 0–1–2–3; 0 = strongly agree, 1 = agree moderately, 2 = agree slightly, 3 = strongly disagree). According to the recommended cutoff point of PWI-SF, those scoring 27 points or more were placed in the high-risk group, those scoring 9 to 26 points were placed in the potential stress group, and those scoring 8 points or less were placed in the healthy group. Cronbach’s α coefficient was 0.91 for subjects in the present study.

#### 2.2.3. Lifestyle

Employees were asked to record current smoking, drinking, sleeping, and exercise habits on the self-administered questionnaire using the following response options: smoking status was classified into “smoker” and “non-smoker”; drinking status divided into “<1 (Month)”, “1 (Month)”, “2–4 (Month)”, “2≤ (Week)”; sleeping status (hours per day) was categorized as “<6”, “6–8”, “8<”; and exercise status (hours per week) was categorized as “none”, “<1”, “1–2”, “2<”. The exercise time category was based on the minimum time of one hour for health promotion through walking in daily life [28], and the lifestyle category also referred to the Korea National Statistical Office standards [39].

### 2.3. Statistical Analysis

The data used for statistical analysis, as well as the missing data, were tested for homogeneity. The sociodemographic characteristics of employees were measured using frequency, percentage, mean, and standard deviation, and then ANOVA and Scheffe tests were conducted. The mean of the PJ and the IJ by sociodemographic characteristics and lifestyle factors was calculated. We conducted multiple logistic regression analyses to estimate association of PJ and IJ with the prevalence odds ratios and 95% confidence intervals of the high risk to PW. Subsequently, we adjusted for gender, age, education, type of employment, type of work, and additionally, for smoking, drinking, and sleeping habits. These analyzed values were stratified by the regular exercise category. Statistical analyses were performed using SPSS 21.0 software (IBM, Armonk, NY, USA).

## 3. Results

### 3.1. Procedural Justice and Interactional Justice by Sociodemographic Characteristics

The mean scores and standard deviations of PJ and IJ by sociodemographic are shown in Table 1. We observed gender differences in the results of PJ (3.62 in men and 3.29 in women) and IJ (3.66 in men and 3.37 in women). In reference to the age corresponding to the PJ mean, aged 50 and over groups were the highest and groups 30–39 were the lowest. The IJ mean according to age items was not significant. In the educational category, the OJ mean for “more than 12 years” was higher than that of “less than 12 years”. The differences in the OJ means based on type of employment and type of work item were not significant. The means of PJ and IJ were the highest in small-sized enterprises and lowest in medium-sized enterprises.

### 3.2. Procedural Justice and Interactional Justice by Lifestyle Factors

Table 2 presents the mean scores of PJ and IJ by lifestyle. In reference to smoking and the PJ mean, “smoker” with 3.69 was higher than “non-smoker” with 3.42 (*p*-value: 0.014), but the difference between the IJ means was not significant. The difference between the OJ means in the drinking items was also not significant. In the sleep category, employees answering ‘<6 hours’ reported the highest PJ and those answering ‘8< hours’ reported the highest IJ. A significant clear gradient of the OJ mean scores was found for the regular exercise items from the ‘none’ group (PJ: 3.31, IJ: 3.27) to the ‘2<‘ group (PJ: 3.72, IJ: 3.8). In addition, in the PJ and IJ mean differences according to PW, a clear gradient was found from the ‘high-risk group’ with 2.99 and 2.94 to the ‘healthy group’ with 4.44 and 4.47, respectively.

### 3.3. Association of Procedural Justice and Interactional Justice with Psychological Well-Being by Regular Exercise among Korean Employees

Table 3 and Table 4 show the main effect of PJ and IJ on the high risk to PW, and the association of PJ and IJ with the prevalence odds ratios and 95% confidence intervals of the high risk to PW. Model 1 was not adjusted, Model 2 adjusted sociodemographic characteristics, and Model 3 further adjusted its lifestyle. The prevalence odds ratios of the high risk to PW were less than 1, and these patterns remained unchanged after adjusting for sociodemographic characteristics and lifestyle factors. In model 3, main effects of PJ and IJ were 0.43 and 0.41, respectively. The prevalence odds ratios of the high risk to PW were stratified by the regular exercise category. A gradient of the effects of OJ on the prevalence odds ratios of a high risk to PW stratified by regular exercise was founded. The odds ratio of the ‘1–2 h’ group was lowest and ‘2<‘ group was not significant in Model 3.

## 4. Discussion

This study compared the mean of organizational justice according to sociodemographic and lifestyle factors and examined the association between OJ and PW by regular exercise (hours per week) in Korean employees.

In our study, the difference in the OJ mean by gender was higher for men than for women. A study conducted in Taiwan has shown that female employees in large enterprises experienced greater exposure to an unfair organizational environment [13]. However, in the studies from Japan and Taiwan [6,13], since the difference in the mean score of OJ between male and female employees was not significant, a comparison of OJ by gender needs to be studied further. The level of perceived OJ by the older employees group was higher than that of younger groups. The result was consistent with the Taiwan study and might be interpreted as being due to the selection of older employees who are not satisfied with organization and poor health in the labor market [13].

In our study, the group with higher education had a high level of perceived OJ. Most of the group with less education held lower ranking positions than the highly educated groups in the workplace. The Taiwan study presents data showing that low-ranking employees were more likely to experience job injustice, because they have fewer opportunities to participate in decision making [13]. However, the study in the UK did not find an association between employment grade and PJ levels [40], and the study among Finnish employees showed a lower PJ in the higher socioeconomic groups. Therefore, the relationship between the level of education and perceived OJ should be studied further [9,36]. The small enterprises showed the highest level of OJ. This finding was consistent with the Taiwan study, which compared OJ by the size of enterprises in the private sector and showed a clearly increasing gradient in the level of OJ from large to small enterprises. The levels of OJ by size of enterprise could be influenced by social factors, including industrial relations, labor legislation, and organizational policy [13].

Within the category of smoking status, the mean score of the OJ was higher in smokers than in non-smokers, but that in the japan study was not significant [12]. Effects of PJ on psychological distress were greater for smokers than non-smokers in the japan study [12]. We suspected that employees would smoke to cope with unfair organizational environments rather than structural changes, such as administrative, institutional, and legal improvements. In a previous study, although smokers showed lower levels of stress tolerance compared to non-smokers, they described the smoking to be temporary relief of distress relating anxiolytic and antidepressant effects [41]. Furthermore, Japanese people sometimes use smoking as a communication tool. Utilizing this cultural practice could improve the relationship between supervisors and employees, and thus raise the level of IJ [12]. The practice of using smoking as a communication tool is likely to apply to Koreans as well.

Those employees who slept for less than six hours showed the lowest level of IJ. The result was consistent with the findings from the Japan study revealing that short sleep durations were related to insomnia and low IJ was a significant risk factor for insomnia [11]. In a study examining the relationship between alcohol disorders and job stress, occupational stress was highlighted as a potential risk factor for alcohol abuse and dependence [42]. The relationship between organizational justice and alcohol abuse needs to be studied further.

A very clear gradient was observed between exercise and OJ, and the level differential of OJ between the exercise group and the non-exercise group was greater for IJ than for PJ. Previous studies have demonstrated the effects of exercise on psychological and mental health factors; physical activity could reduce symptoms of depression [26]. In addition, sedentary behavior in the workplace is associated with increased psychological distress and hopelessness [20]. Participating in sports reduces suicidal ideation by promoting self-esteem and social interaction [43]. The positive effects of regular exercise seem to lead to a positive effect to perceived level of OJ, which is closely related to mental and physical health.

The level of perceived OJ was significantly higher in the healthy PW group. The Japan study showed that the lower the OJ, the higher the odds ratio of serious psychological distress [12]. These findings might be interpreted that a high level of perceived OJ could stay good mental health and PW. Low OJ was significantly associated with prevalence odds ratios of the high risk to PW. This result could be interpreted as being consistent with the Japan study that showed that low OJ was associated with poor mental health [8,9,10,44]. 

In our study, the difference in values between the <1 h group and the 1–2 h group was greater in IJ than in PJ in effects of OJ on the high risk to PW stratified by regular exercise category. Hassmen [24] demonstrated that those who exercised more frequently had a significantly stronger sense of coherence, which can be defined as the extent of feeling generally that life is comprehensible, manageable, and meaningful [45], and were considered to be more socially integrated than those who exercised less frequently or never exercised. The strengthening of sociality by having more regular exercise hours could be related to IJ, which explains whether supervisors and employees treat each other with respect and consideration, rather than to PJ, which explains whether the opinions of the affected employees are correctly applied to the decision-making process.

When the odds ratio was stratified by regular exercise hours, our study showed the gradients of the effects of OJ on the prevalence odds ratios of the high-risk group to PW. Furthermore, those who exercise at twice a week showed higher levels of a sense of coherence [24]. The previous study showed that people who have a strong sense of coherence could successfully manage their stress and maintain their health, while people who have a weak sense of coherence tend to be more vulnerable to health problems [10]. Answers of two or more hours a week of exercise in the effects of OJ on the prevalence odds ratios of the high risk to PW stratified by regular exercise were not significant. The Michigan study reported that those who exercise more frequently tend to be less inclined to suppress their anger and hostility in comparison with those who exercise less frequently [46]. Based on this evidences, the association between OJ and PW was strongest in the group that exercised 1 to 2 h a week, and the differences were also stronger in IJ than in PJ.

### Limitations and Strengths

First, the subjects sample size of our study was small. Thus, generalization of the findings in Korean employees should be done with caution. Second, the original version instrument and the Korean version measuring OJ lack a subscale for distributive justice. Third, because this study used a cross-sector design, exposures and results were evaluated simultaneously. Fourth, factors related to the association between OJ and PW should be considered in relation to not only exercise but also other social factors. Finally, since we used self-administered questionnaires to examine lifestyle, OJ, and PW, these were likely to be affected by the personality and psychological factors of the individual completing the questionnaire. Despite these listed limitations, our study used reliable instruments to measure organizational justice and psychological well-being. Also, to the best of our knowledge this study was the first to measure organizational justice as a psychosocial predictor of health in Korea. In addition, this study was investigated by classifying by size of the enterprise to help generalize. The results from the survey may be applicable to small, medium, and large enterprises.

## 5. Conclusions

The ‘OJ’ might be a protective factor for ‘high risk to PW’ in Korean employees. The lowest odds ratio was observed in a group that exercised for 1–2 h (hours per week) in association between OJ and high risk to PW. The underlying mechanisms for the observed stronger associations in a group that regularly exercises than in a group that does not exercise should be further studied.

## Figures and Tables

**Table 1:** Mean values of procedural justice and interactional justice by sociodemographic characteristics.

Characteristics	N (%)	Procedural Justice Mean (SD ^†^)	*p*-Value	Interactional Justice Mean (SD ^†^)	*p*-Value
Total	376 (100)	3.49 (0.89)		3.54 (0.93)	
Gender			0.000		0.003
Men	219 (58.2)	3.62 (0.92)		3.66 (0.95)	
Women	157 (41.8)	3.29 (0.81)		3.37 (0.87)	
Age			0.028		0.055
20–29	83 (22.1)	3.54 (0.79)		3.50 (0.85)	
30–39	150 (39.9)	3.38 (0.85)		3.46 (0.93)	
40–49	79 (21.0)	3.41 (0.99)		3.51 (0.98)	
50≤	64 (17.0)	3.76 (0.92)		3.83 (0.92)	
Education			0.003		0.005
12≤	75 (19.9)	3.76 (0.88)		3.81 (0.93)	
<12	301 (80.1)	3.42 (0.88)		3.47 (0.92)	
Type of employment			0.801		0.558
Regular	298 (79.3)	3.48 (0.91)		3.53 (0.95)	
Temporary	78 (20.7)	3.51 (0.80)		3.60 (0.86)	
Type of work			0.072		0.115
Daytime	350 (93.1)	3.52 (0.84)		3.57 (0.88)	
Shift work	26 (6.9)	3.03 (1.29)		3.12 (1.37)	
Size of enterprise			0.000		0.003
large (300≤)	142 (37.8)	3.50 (0.94)		3.57 (1.00)	
medium (50–299)	131 (34.8)	3.23 (0.79)		3.30 (0.82)	
small (1–49)	103 (27.4)	3.79 (0.84)		3.81 (0.89)	

^†^ SD = standard deviation.

**Table 2:** Mean values of procedural justice and interactional justice by lifestyle factors.

Characteristics		Procedural Justice Mean (SD)	*p*-Value	Interactional Justice Mean (SD)	*p*-Value
	N (%)				
Total	376 (100)	3.49 (0.89)		3.54 (0.93)	
Smoking			0.014		0.142
Smoker	89 (23.7)	3.69 (0.88)		3.67 (0.90)	
Non-smoker	287 (76.3)	3.42 (0.88)		3.50 (0.94)	
Drinking			0.10		0.36
<1 (Month)	93 (24.7)	3.35 (0.84)		3.45 (0.91)	
1 (Month)	61 (16.2)	3.36 (1.10)		3.42 (1.09)	
2–4 (Month)	136 (36.2)	3.60 (0.86)		3.61 (0.89)	
2≤ (Week)	86 (22.9)	3.55 (0.80)		3.62 (0.87)	
Sleeping (hours per day)			0.00		0.00
<6	143 (38)	3.50 (0.94)		3.50 (1.00)	
6–8	221 (58)	3.49 (0.85)		3.57 (0.88)	
8<	12 (4)	3.32 (0.95)		3.55 (0.94)	
Exercise (hours per week)			0.01		0.001
None	94 (25.0)	3.31 (0.91)		3.27(0.96)	
<1	126 (33.5)	3.40 (0.96)		3.49 (1.01)	
1–2	85 (22.6)	3.61 (0.82)		3.70 (0.78)	
2<	71 (18.9)	3.72 (0.73)		3.8 (0.79)	
Psychological well-being			0.00		0.00
High-risk group (27≤)	88 (23.4)	2.99 (0.83)		2.94 (0.88)	
Potential-stress group (9~26)	256 (68.1)	3.54 (0.82)		3.63 (0.85)	
Healthy group (≤8)	32 (8.5)	4.44 (0.65)		4.47 (0.61)	

**Table 3:** Association of procedural justice with psychological well-being by regular exercise among Korean employees.

Procedural Justice	N	Prevalence Odds Ratio (95% Confidence Interval)		
		Model 1	Model 2	Model 3
Main effect	376	0.41 (0.30–0.55)	0.40 (0.28–0.56)	0.43 (0.30–0.62)
Exercise				
None	94	0.66 (0.40–1.07)	0.59 (0.33–1.04)	0.70 (0.37–1.33)
<1 h	126	0.33 (0.19–0.57)	0.31 (0.17–0.58)	0.31 (0.15–0.64)
1–2 h	85	0.30 (0.13–0.69)	0.27 (0.10–0.71)	0.27 (0.10–0.76)
2<	71	0.38 (0.12–1.18)	0.42 (0.09–2.00)	0.13 (0.008–2.21)

Model 1: Without any adjustment. Model 2: Adjusted for gender, age, education, type of employment, type of work. Model 3: Additionally adjusted for smoking, drinking, sleeping.

**Table 4:** Association of interactional justice with psychological well-being by regular exercise among Korean employees.

Interactional Justice	N	Prevalence Odds Ratio (95% Confidence Interval)		
		Model 1	Model 2	Model 3
Main effect	376	0.37 (0.27–0.50)	0.38 (0.27–0.52)	0.41 (0.29–0.58)
Exercise				
None	94	0.52 (0.32–0.85)	0.46 (0.26–0.81)	0.53 (0.28–0.99)
<1 h	126	0.39 (0.24–0.63)	0.40 (0.24–0.67)	0.42 (0.24–0.75)
1–2 h	85	0.24 (0.10–0.56)	0.19 (0.71–0.51)	0.19 (0.06–0.57)
2<	71	0.27 (0.08–0.86)	0.40 (0.10–1.49)	0.42 (0.08–2.00)

Model 1: Without any adjustment. Model 2: Adjusted for gender, age, education, type of employment, type of work. Model 3: Additionally adjusted for smoking, drinking, sleeping.
